# Evaluation of Microbial Transformation of 10-deoxoartemisinin by UPLC-ESI-Q-TOF-MS^E^

**DOI:** 10.3390/molecules24213874

**Published:** 2019-10-28

**Authors:** Yue Bai, Dong Zhang, Peng Sun, Yifan Zhao, Xiaoqiang Chang, Yue Ma, Lan Yang

**Affiliations:** 1 Institute of Chinese Materia Medica, China Academy of Chinese Medical Sciences, Beijing 100700, China; byyue_2019@126.com (Y.B.); dzhang@icmm.ac.cn (D.Z.); psun@icmm.ac.cn (P.S.); zyfan_666@163.com (Y.Z.); 19937381620@163.com (X.C.); 2 Artemisinin Research Center, China Academy of Chinese Medical Sciences, Beijing 100700, China

**Keywords:** 10-deoxoartemisinin, microbial transformation, hydroxylation ability, UPLC-ESI-Q-TOF-MS^E^

## Abstract

10-deoxoartemisinin is a semisynthetic derivative of artemisinin that lacks a lactone carbonyl group at the 10-position, and has stronger antimalarial properties than artemisinin. However, 10-deoxoartemisinin has limited utility as a therapeutic agent because of its low solubility and bioavailability. Hydroxylated 10-deoxoartemisinins are a series of properties-improved derivatives. Via microbial transformation, which can hydroxylate 10-deoxoartemisinin at multiple sites, the biotransformation products of 10-deoxoartemisinin have been investigated in this paper. Using ultra-performance liquid chromatography-electrospray ionization-quadrupole time-of-flight mass spectrometry (UPLC-ESI-Q-TOF-MS^E^) combined with UNIFI software, products of microbial transformation of 10-deoxoartemisinin were rapidly and directly analyzed. The hydroxylation abilities of nine microorganisms were compared using this method. All of the microorganisms evaluated were able to hydroxylate 10-deoxoartemisinin, and a total of 35 hydroxylated products were identified. These can be grouped into dihydroxylated 10-deoxoartemisinins, monohydroxylated 10-deoxoartemisinins, hydroxylated dehydrogenated 10-deoxoartemisinins, and hydroxylated hydrogenated 10-deoxoartemisinins. *Cunninghamella echinulata* and *Cunninghamella blakesleeana* are able to hydroxylate 10-deoxoartemisinin, and their biotransformation products are investigated here for the first time. *Cunninghamella elegans* CICC 40250 was shown to most efficiently hydroxylate 10-deoxoartemisinin, and could serve as a model organism for microbial transformation. This method could be used to generate additional hydroxylated 10-deoxoartemisinins for further research.

## 1. Introduction

Artemisinin is a first-line antimalarial agent, which is first derived from Chinese herb by Professor Tu [1]. 10-deoxoartemisinin is a bioactive derivative of artemisinin, first prepared by the simple transformation in 1990 [2]. It lacks the C-10 lactone carbonyl group of artemisinin, but retains its antimalarial endoperoxide activity [3]. Compared with artemisinin, the derivate was eight times more active against multidrug-resistant malaria in vitro [2]. However, poor solubility and bioavailability are the barriers for further application in the clinic. Moreover, 10-deoxoartemisinin also has impressive antitumor and antiangiogenesis activities [4,5,6]. As a part of our ongoing projects [7,8,9,10], novel 10-deoxoartemisinin derivatives with improved the properties are attractive to develop for further research. Hydroxylation, as the main modification route, could not only improve chemical properties, but provide candidates of novel drug development.

**Figure 1:**
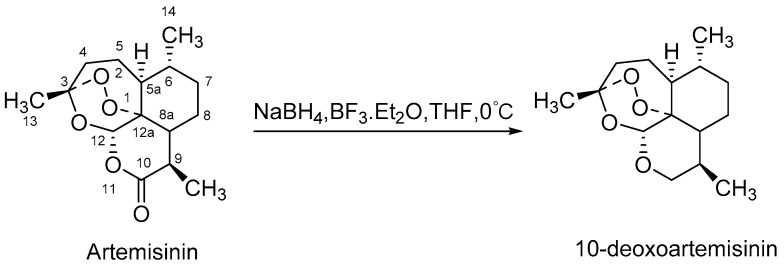
Scheme synthesis of 10-deoxoartemisinin.

In recent years, microbial transformation has become an efficient tool to generate novel active products. Microorganisms can achieve predetermined selectivity to add functional groups in an environment-friendly manner because of their abundant cytochrome P450 enzymes [11,12,13]. Several fungi have been reported to exhibit particularly strong hydroxylation capabilities, including *Cunninghamella*, *Aspergillus, Mucor*, etc. [14,15,16]. Up to now, five microbial transformation products of 10-deoxoartemisinin from four strains have been documented. These include 5β-hydroxy-10-deoxoartemisinin from *Mucor ramannianus* [17], 4α-hydroxy-1, 10-deoxoartemisinin and 7β-hydroxy-10-deoxoartemisinin from both *Mucor ramannianus* and *Cunninghamella elegans* [18], 15-hydroxy-10-deoxoartemisinin from *Aspergillus Niger* [19], and a 13-carbon rearranged product from *Aspergillus ochraceus* [20], none of which were reported to be bioactive. Even though, 10-deoxoartemisinin provides an excellent biotransformation substrate for the production of new potentially antimalarial active, and there remains a need for more exploited. The scheme synthesis of 10-deoxoartemisinin is shown in Figure 1.

Different microbial strains may use distinct pathways to accomplish biotransformation, making a rapid and direct method to evaluate hydroxylation products is imperative. Here, ultra-performance liquid chromatography-electrospray ionization-quadrupole time-of-flight mass spectrometry (UPLC-ESI-Q-TOF-MS^E^) is established as the method to predict the hydroxylation of 10-deoxoartemisinin by nine different microorganisms. These microorganisms studied in this research were *Cunninghamella elegans* CICC 40250 (**MT1**), *Mucor circinelloides* CGMCC 3.3421 (**MT2**), *Cunninghamella elegans* CGMCC 3.4832 (**MT3**), *Cunninghamella echinulata* CGMCC 3.4879 (**MT4**), *Mucor circinelloides* CGMCC 3.49 (**MT5**), *Cunninghamella echinulata* CGMCC 3.5771 (**MT6**), *Cunninghamella blakesleeana* CGMCC 3.5802 (**MT7**), *Cunninghamella blakesleeana* CGMCC 3.910 (**MT8**), and *Cunninghamella elegans* ATCC 9245 (**MT9**), which have all been reported to perform hydroxylation reactions [21,22,23,24,25]. The biotransformation products of 10-deoxoartemisinin were analyzed and identified using UNIFI software. Among these organisms, *Cunninghamella elegans* CICC 40250 (**MT1**) exhibited the best hydroxylation profile, and holds promise for generating novel bioactive hydroxylated products for further research.

## 2. Results

### 2.1. Identification of 10-deoxoartemisinin

**Figure 2:**
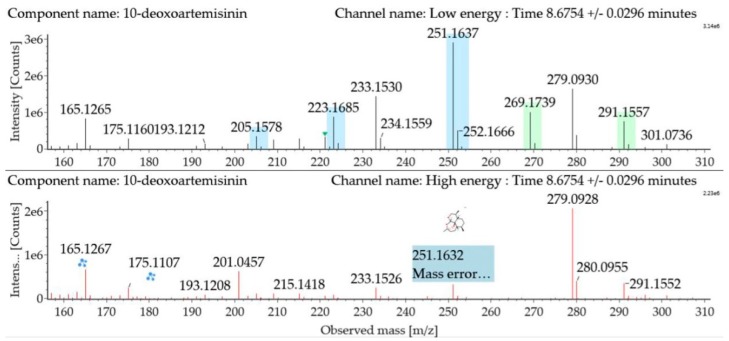
Ultra-performance liquid chromatography-electrospray ionization-quadrupole time-of-flight mass spectrometry (UPLC-ESI-Q-TOF-MS^E^) spectra and fragmentation diagram of 10-deoxoartemisinin.

**Figure 3:**
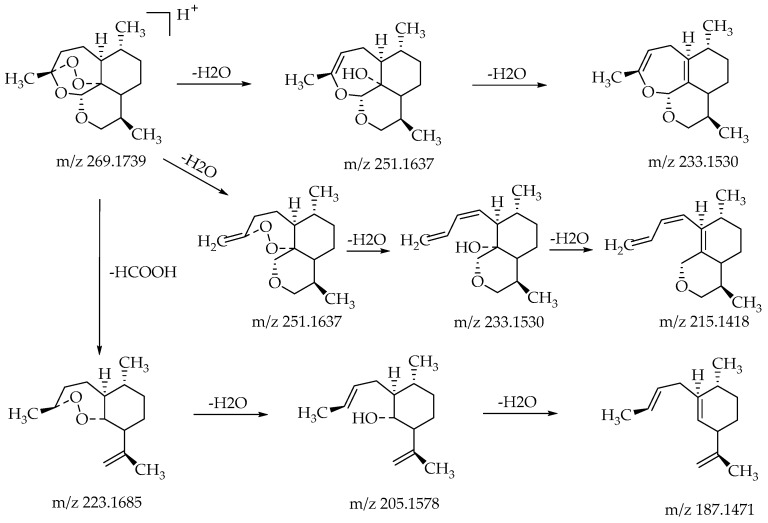
Proposed fragmentation pathways of 10-deoxoartemisinin.

The substrate 10-deoxoartemisinin was used to establish a reference fragmentation pattern. Its molecular ions [M + H]^+^ and [M + Na]^+^ were observed at *m*/*z* 269.1739 and *m*/*z* 291.1557. The fragment ions at *m/z* 251.1637, 233.1530, and 215.1418 were generated by the successive loss of water from *m/z* 269.1739. The fragment ion at *m*/*z* 223.1685 represented the loss of CO from *m*/*z* 251.1637 or the loss of HCOOH from *m*/*z* 269.1739. The fragment ion at *m/z* 205.1578 resulted from the loss of water from *m*/*z* 223.1685. Mass spectra and the fragmentation diagram of 10-deoxoartemisinin are shown in Figure 2, and the proposed fragmentation pathways are shown in Figure 3.

### 2.2. Identification of Microbial Transformation Products

**Figure 4:**
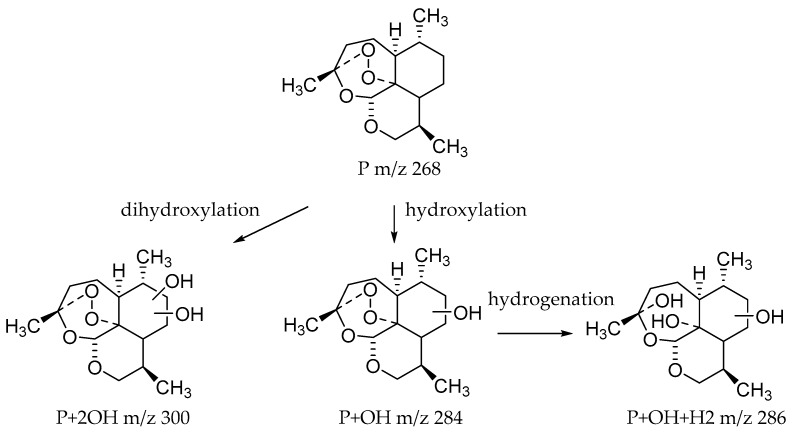
Structures of predicted transformation products.

Microbial transformation products were identified using the characteristic mass spectrometric behavior of 10-deoxoartemisinin, including parent ions, internal cleavage of the ion, and retention times. Altogether, 35 transformation products were identified; primarily dihydroxylated 10-deoxoartemisinin, monohydroxylated 10-deoxoartemisinin, hydroxylated dehydrogenated 10-deoxoartemisinin, and hydroxylated hydrogenated 10-deoxoartemisinin. While all strains tested were able to produce hydroxylated 10-deoxoartemisinins, **MT1** produced the most derivatives, with 18 distinct products. The 18 hydroxylated products from **MT1** all had a peroxide bridge. These results demonstrate that **MT1** is an ideal microorganism model to obtain hydroxylated 10-deoxoartemisinin for further research. Meanwhile, the biotransformation products from *Cunninghamella echinulata* and *Cunninghamella blakesleeana* are first to be investigated. The identified microbial transformation products are shown in Table 1, and the structures of predicted transformation products are shown in Figure 4.

**Figure 5:**
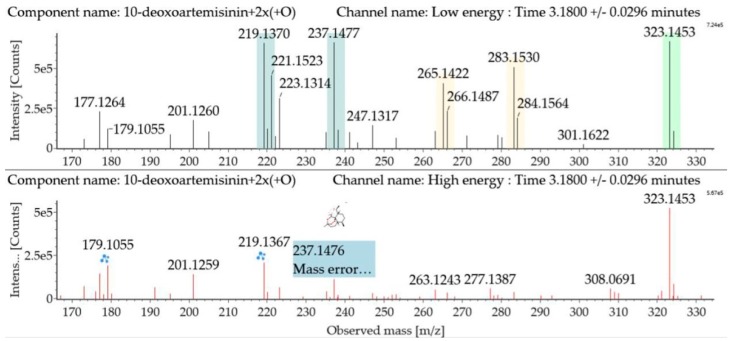
UPLC-ESI-Q-TOF-MS^E^ spectra and the fragmentation diagram of dihydroxylated 10-deoxoartemisinin.

Dihydroxylated 10-deoxoartemisinin transformation products were detected between 2.83 and 4.41 min, and the parent ions were detected at *m*/*z* 323 ([M + Na]^+^) and *m*/*z* 301 ([M + H]^+^). The products were identified by comparing their parent ions to 10-deoxoartemisinin, which showed a series of fragment ions resulting from H_2_O and CO loss. The dihydroxylated products were more likely to lose two water molecules compared with 10-deoxoartemisinin, and the fragment ions at *m*/*z* 265 and 247 were generated by successive water loss from *m*/*z* 283. The fragment ion at *m*/*z* 237 was generated by CO loss from *m*/*z* 265 or HCOOH loss from *m*/*z* 283. The fragment ion at *m*/*z* 219 was generated by water loss from *m*/*z* 237. Mass spectra and the fragmentation diagram for dihydroxylated 10-deoxoartemisinin products are shown in Figure 5. 

**Figure 6:**
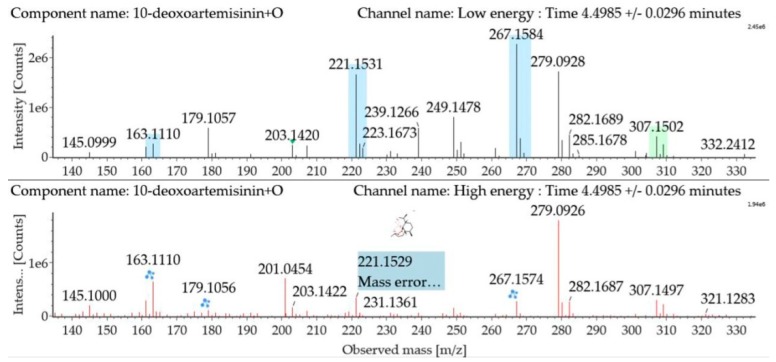
UPLC-ESI-Q-TOF-MS^E^ spectra and the fragmentation diagram of monohydroxylated 10-deoxoartemisinin.

Monohydroxylated 10-deoxoartemisinin transformation products were detected between 3.14 and 6.91 min. The products were identified by comparing their parent ions to 10-deoxoartemisinin, with a 16 Da mass shift attributed to an oxygen atom. The molecular ion at *m*/*z* 307 ([M + Na]^+^) was observed. The fragment ion at *m*/*z* 267 resulted from water loss from *m*/*z* 285. The fragment ions at *m*/*z* 249 and *m*/*z* 231 were generated by water loss from *m*/*z* 267 and *m*/*z* 249, respectively. The fragment ion at *m*/*z* 221 resulted from CO loss from *m*/*z* 249. Mass spectra and the fragmentation diagram for monohydroxylated 10-deoxoartemisinin products are shown in Figure 6. 

**Figure 7:**
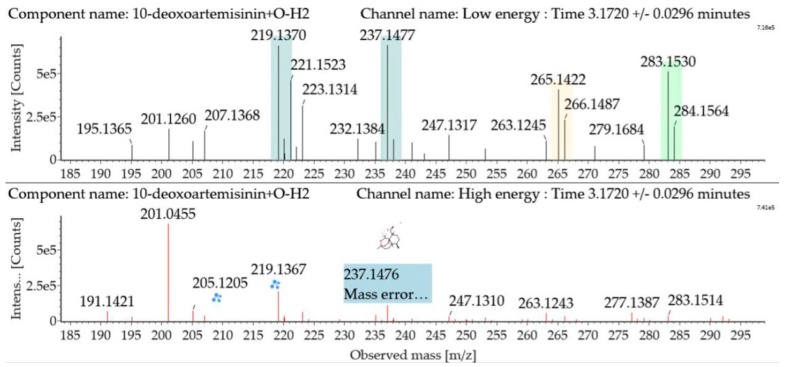
UPLC-ESI-Q-TOF-MS^E^ spectra and the fragmentation diagram of hydroxylated dehydrogenated 10-deoxoartemisinin.

Hydroxylated dehydrogenated 10-deoxoartemisinin products were detected between 2.83 and 5.57 min. The molecular ion at *m*/*z* 283 ([M + H]^+^) was observed. Consistent with 10-deoxoartemisinin, these products showed a series of fragment ions resulting from the loss of H_2_O, CO, or HCOOH. Mass spectra and the fragmentation diagram for hydroxylated dehydrogenated 10-deoxoartemisinin products are shown in Figure 7. 

**Figure 8:**
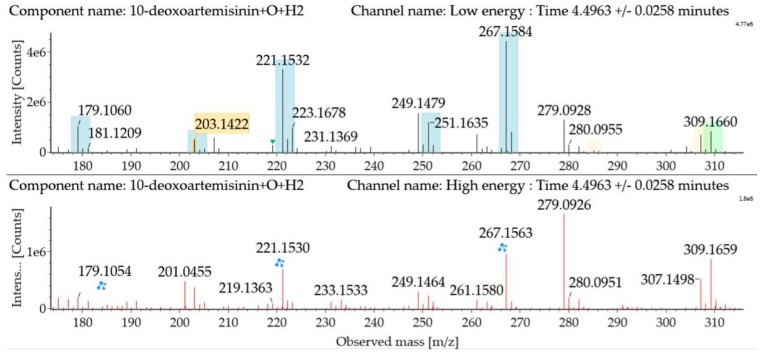
UPLC-ESI-Q-TOF-MS^E^ spectra and the fragmentation diagram of hydroxylated hydrogenated 10-deoxoartemisinin.

Hydroxylated hydrogenated 10-deoxoartemisinin products were detected at about 4.50 min. The fragment ions were similar to monohydroxylation products of 10-deoxoartemisinin. The parent ion was detected at *m*/*z* 309 ([M + Na]^+^). Fragment ions at *m*/*z* 249 and 231 were generated by successive water loss from *m*/*z* 267. The fragment ion at *m*/*z* 221 was generated by CO loss from *m*/*z* 249 or HCOOH loss from *m*/*z* 267. The fragment ion at *m*/*z* 203 resulted from water loss from *m*/*z* 221. The mass spectra and the fragmentation diagram for hydroxylated hydrogenated 10-deoxoartemisinin products are shown in Figure 8.

## 3. Discussion

Hydroxylation is the main route for molecular modification and also a primary metabolic reaction in vivo involving artemisinin and its derivatives [20,26]. Hydroxylated 10-deoxoartemisinins are a series of products that have a peroxide bridge and may retain antimalarial activity, with better physiological solubility compared with unmodified 10-deoxoartemisinin. Meanwhile, these hydroxylated compounds provide the new reactive site to link another functional group for developing a novel active drug.

Microbial transformation is an increasingly popular method used to modify small molecules, particularly adept at generating hydroxylated derivatives, because many enzymes catalyze hydroxylation reactions, and a variety of microorganisms can be used in this method. The microbial transformation was used in this study to find out novel hydroxylated 10-deoxoartemisinins. The hydroxylation abilities of nine microorganisms from the genera *Cunninghamella* and *Mucor* was evaluated, because previous studies have shown that species in these two genera are rich in hydroxylases [27]. Additionally, *C. elegans*, *C. echinulata*, *C.blakesleeana,* and *M. circinelloides* have been successfully employed to hydroxylate other substrates [28,29,30]. The hydroxylation ability of these microorganisms was estimated by identifying and comparing their hydroxylation products. A total of 35 hydroxylated products were identified from the nine microorganisms. 

Strains **MT1-MT9** produced 18, 1, 4, 15, 1, 9, 14, 7, and 3 distinct hydroxylated 10-deoxoartemisinins, respectively. These hydroxylated 10-deoxoartemisinins were categorized as ehydroxylated 10-deoxoartemisinins, monohydroxylated 10-deoxoartemisinins, hydroxylated dehydrogenated 10-deoxoartemisinins, and hydroxylated hydrogenated 10-deoxoartemisinins. Strains **MT1**, **MT4**, **MT6,** and **MT7** had the best hydroxylation abilities, generating more than ten hydroxylated products each, with products from all four of the above categories. Strains **MT2** and **MT5** showed only weak hydroxylation, producing only one monohydroxylated 10-deoxoartemisinin each. In addition, strains **MT3** and **MT9** only produced monohydroxylated 10-deoxoartemisinins. **MT1** (*C. elegans* CICC 40250) exhibited the best hydroxylation ability, with 18 hydroxylated products identified. **MT1** could serve as a model organism for this process and could also produce novel hydroxylated 10-deoxoartemisinins. Overall, *Cunninghamella* hydroxylates 10-deoxoartemisinin better than *Mucor*. 

The rapid and direct analysis method developed in this paper is significant for efficiently screening numerous of Microorganisms transformation. The results could be the guidance for further isolation research to obtain more derivates. This work is foundational for producing novel, potentially bioactive, hydroxylated 10-deoxoartemisinins. 

## 4. Materials and Methods

### 4.1. Materials and Reagents

10-deoxoartemisinin was synthesized from artemisinin, as previously described [2]. *M. circinelloides* CGMCC 3.3421, *M. circinelloides* CGMCC 3.49, *C. blakesleeana* CGMCC 3.910 and 3.5802, *C. echinulata* CGMCC 3.5771, *C. echinulata* 3.4879, and *C. elegans* CGMCC 3.4832 were obtained from the China General Microbiological Culture Collection Center (Beijing, China). *C. elegans* CICC 40250 was obtained from the China Center of Industrial Culture Collection (Beijing, China). *C. elegans* ATCC 9245 was obtained from the American Type Culture Collection (Virginia, United States). Acetonitrile and methanol were purchased from Fisher (Geel, Belgium), and formic acid, EtOAc (ethyl acetate), and other chemicals used in this research were purchased from Beijing Chemical Works (Beijing, China). 

### 4.2. Culture and Biotransformation Procedure

Culture and biotransformation experiments were conducted in a medium composed of 20 g Sabouraud Dextrose Broth (Oxoid, Basingstoke, UK), 10 g peptone (Solarbio, Beijing, China), 15 g sucrose (Solarbio, Beijing, China), and 1000 mL deionized water [31,32].

Two-stage fermentation was employed in this study [33,34]. The substrate was dissolved in MeOH at a concentration of 25 mg/mL, and 2 mL of the solution was added into each flask after the second fermentation stage, producing a 10-deoxoartemisinin concentration of 0.5 mg/mL. Cultures were incubated at 28 °C and shaken at 180 rpm for 14 days. Mycelia and broth were then separated by filtration. The filtrate was extracted with EtOAc (1:1, *v*/*v*) three times, and the extract was evaporated under vacuum to produce a brown residue, which was dissolved in MeOH for LC-MS analysis.

### 4.3. LC–MS^E^ Conditions and Data Processing

Mass spectrometry conditions were established in previous studies [32]. The UPLC-ESI-Q-TOF-MS^E^ system consisted of a Waters ACQUITY I-class UPLC and Xevo G2-XS Q-TOF Mass Spectrometer (Waters, Manchester, UK). Chromatography was conducted using an Acquity UPLC BEH C_18_ (2.1 mm × 100 mm, 1.7 µm (Waters, Manchester, UK)). The mobile phase consisted of solvent A (H_2_O containing 0.1% formic acid, *v*/*v*) and solvent B (acetonitrile containing 0.1% formic acid, *v*/*v*). The gradient program included three segments: 5%–100% B from 0 to 15 min; 100%–5% B from 15 to 17 min; followed by a 3 min post-run for column equilibration. The flow rate was 0.4 mL/min, and the temperature was 25 °C throughout the analysis. 

The MS was operated in positive ionization mode across a scan range of 50 to 1200 *m*/*z*. The low collision energy was set at 6 eV, and the high collision energy was ramped from 12 to 25 eV. MS^E^ analysis was conducted using multiple reactions monitoring positive-ion electrospray ionization. 

All data processing was performed using UNIFI 1.9 (Waters, Manchester, UK). Components were identified using the following 3D peak detection features: Low-energy limits of 150 and high-energy limits of 20, isotope clustering, and high-to-low energy association within a 0.5 fraction of the chromatographic and drift peak width, with a mass accuracy of ± 2 mDa. The maximum number of allowed fragment ions per match was set to 10.

## 5. Conclusions

This research establishes microbial transformation as a method for obtaining various hydroxylated products. Transformation products from nine microorganisms were identified using UPLC-ESI-Q-TOF-MS^E^, and the hydroxylation ability of the nine microorganisms was evaluated. Transformation products of 10-deoxoartemisinin were categorized as hydroxylated 10-deoxoartemisinins, hydroxylated dehydrogenated 10-deoxoartemisinins, and hydroxylated hydrogenated 10-deoxoartemisinins. **MT1** (*C. elegans* CICC 40250) exhibited the best hydroxylation ability among the selected microorganisms, and could be used as a transformation model to prepare hydroxylated 10-deoxoartemisinins for further research. Additionally, this study was the first to investigate the transformation products of 10-deoxoartemisinin from *C. echinulata* and *C. blakesleeana*.

## Figures and Tables

**Table 1:** Summary of microbial transformation products of 10-deoxoartemisinin.

NO.	Component Name	Formula	RT (min)	Major Fragments	MT1	MT2	MT3	MT4	MT5	MT6	MT7	MT8	MT9
1	P+2O	C_15_H_24_O_6_ + Na^+^	3.18	323, 283, 265, 247, 237, 219	+			+		+			
2	P+2O	C_15_H_24_O_6_ + Na^+^	3.62	323, 283, 265, 237, 221, 203	+					+			
3	P+2O	C_15_H_24_O_6_ + Na^+^	3.72	323, 283, 265, 247, 237, 219, 201							+		
4	P+2O	C_15_H_24_O_6_ + Na^+^	2.83	323, 300, 283, 265, 247, 237, 219				+		+	+		
5	P+2O	C_15_H_24_O_6_ + Na^+^	2.94	323, 283, 265, 249, 247, 237, 219								+	
6	P+2O	C_15_H_24_O_6_ + Na^+^	4.04	323, 283, 265, 249, 231, 221, 219						+			
7	P+2O	C_15_H_24_O_6_ + Na^+^	4.15	323, 283, 265, 247, 237, 219	+						+	+	
8	P+2O	C_15_H_24_O_6_ + Na^+^	4.41	339, 301, 283, 265, 247, 237, 219				+					
9	P+O	C_15_H_24_O_5_ + Na^+^	3.97	323, 307, 267, 249, 231, 221, 203			+					+	+
10	P+O	C_15_H_24_O_5_ + Na^+^	4.02	307, 283, 265, 249, 221, 303	+			+			+		
11	P+O	C_15_H_24_O_5_ + Na^+^	3.14	307, 285, 267, 249, 231, 221, 203	+			+		+			
12	P+O	C_15_H_24_O_5_ + Na^+^	4.97	307, 283, 267, 249, 239, 221, 203	+			+					
13	P+O	C_15_H_24_O_5_ + Na^+^	4.50	307, 285, 267, 249, 239, 221, 203	+					+	+	+	+
14	P+O	C_15_H_24_O_5_ + Na^+^	4.51	307, 267, 249, 239, 221, 203		+	+	+					
15	P+O	C_15_H_24_O_5_ + Na^+^	4.26	307, 267, 249, 239, 221, 203	+			+			+	+	
16	P+O	C_15_H_24_O_5_ + Na^+^	4.36	285, 267, 249, 239, 221, 203	+						+		
17	P+O	C_15_H_24_O_5_ + Na^+^	4.69	307, 283, 265, 249, 231, 221, 203	+								
18	P+O	C_15_H_24_O_5_ + Na^+^	5.40	307, 283, 267, 249, 221, 203	+		+	+	+	+	+	+	+
19	P+O	C_15_H_24_O_5_ + Na^+^	6.91	323, 307, 267, 249, 231, 221, 203			+						
20	P+O-2H	C_15_H_22_O_5_ + H^+^	3.17	283, 265, 247, 237, 221, 219	+			+		+	+		
21	P+O-2H	C_15_H_22_O_5_ + H^+^	5.57	305, 283, 265, 237, 221, 219	+			+					
22	P+O-2H	C_15_H_22_O_5_ + H^+^	3.61	283, 265, 237, 221, 203	+								
23	P+O-2H	C_15_H_22_O_5_ + H^+^	3.67	305, 283, 265, 247, 237, 219				+					
24	P+O-2H	C_15_H_22_O_5_ + H^+^	2.83	283, 265, 247, 237, 219				+			+		
25	P+O-2H	C_15_H_22_O_5_ + H^+^	2.93	283, 265, 247, 237, 219							+	+	
26	P+O-2H	C_15_H_22_O_5_ + H^+^	3.07	305, 265, 247, 237, 219									
27	P+O-2H	C_15_H_22_O_5_ + H^+^	3.72	283, 265, 247, 237, 219, 201							+		
28	P+O-2H	C_15_H_22_O_5_ + H^+^	4.17	305, 283, 265, 247, 237				+					
29	P+O-2H	C_15_H_22_O_5_ + H^+^	4.16	283, 265, 247, 237, 219, 201						+			
30	P+O-2H	C_15_H_22_O_5_ + H^+^	3.37	283, 265, 247, 237, 219	+								
31	P+O-2H	C_15_H_22_O_5_ + H^+^	4.97	305, 283, 265, 247, 237, 219	+								
32	P+O-2H	C_15_H_22_O_5_ + H^+^	4.50	305, 285, 267, 249, 231, 221, 203							+		
33	P+O-2H	C_15_H_22_O_5_ + H^+^	4.41	283, 265, 247, 237, 219	+								
34	P+O-2H	C_15_H_22_O_5_ + H^+^	4.65	305, 283, 265, 249, 237, 231, 219	+								
35	P+O+2H	C_15_H_26_O_5_ + Na^+^	4.50	309, 285, 267, 249, 231, 221, 203				+			+		
Total hydroxylation products					18	1	4	15	1	9	14	7	3

P represents 10-deoxoartemisinin.

## References

[1] Collaboration Research Group for Qinghaosu (1977). A new sesquiterpene lactone—qinghaosu. KexueTongbao.

[2] Jung M., Li X., Bustos D.A., ElSohly H.N., McChesney J.D., Milhous W.K. (1990). Synthesis and Antimalarial Activity of (+)-Deoxoartemisinin. J. Med. Chem..

[3] Jung M., Li X., Bustos D.A., ElSohly H.N., McChesney J.D. (1989). A short and stereospecific synthesis of (+)-deoxoartemisinin and (−)-deoxodesoxyartemisinin. Tetrahedron Lett..

[4] Lee C.H., Hong H., Shin J., Jung M., Shin I., Yoon J., Lee W. (2000). NMR Studies on Novel Antitumor Drug Candidates Deoxoartemisinin and Carboxypropyldeoxoartemisinin. Biochem. Biophys. Res. Commun..

[5] Jung M., Tak J., Chuang W.Y., Park K.-K. (2006). Antiangiogenic activity of deoxoartemisinin derivatives on chorioallantoic membrane. Bioorg. Med. Chem. Lett..

[6] Jung M., Lee S., Ham J., Lee K., Kim H., Kim S.K. (2003). Antitumor Activity of Novel Deoxoartemisinin Monomers, Dimers, and Trimer. J. Med. Chem..

[7] Tu Y.Y. (2011). The discovery of artemisinin (qinghaosu) and gifts from Chinese medicine. Nat. Med..

[8] Tu Y.Y. (2009). Artemisinin and Artemisinin Drugs.

[9] Wang J., Xu C., Liao F.L., Jiang T., Krishna S., Tu Y. (2019). A Temporizing Solution to “Artemisinin Resistance”. N. Engl. J. Med..

[10] Ma Y., Zhu Y.P., Zhang D., Meng Y., Tang T., Wang K., Ma J., Wang J., Sun P. (2019). Eco-friendly decarboxylative cyclization in water: practical access to the anti-malarial 4-quinolones. Green Chem..

[11] Guan S.-L., Wu Y.-X., Sun H., Zhang Y.C., Zhao R.Q., Wang H., Sun L.J., Su L. (2018). Application of microbial transformation technology in the development of Chinese medicine. Microbiol. China..

[12] Chen D.-J., Zhu B.-Q. (2012). Application of microbial transformation in modern pharmaceutical industry. Chin. J. Antibiot..

[13] Niu H.-J., Wang P., Yang G.-E. (2013). Application of Microbial Transformation in Research of Chinese Medicine. Chin. J. Exp. Trad. Med. Formulae..

[14] Zhan J.X., Guo H.Z., Dai J.G., Zhang Y., Guo D. (2002). Microbial transformations of artemisinin by *Cunninghamella echinulata* and *Aspergillus niger*. Tetrahedron Lett..

[15] Zhan Y.L., Liu H., Wu Y.S., Wei P., Chen Z., Williamson J.S. (2015). Biotransformation of artemisinin by *Aspergillus niger*. Appl. Microbiol. Biotechnol..

[16] Parshikov I.A., Miriyala B., Muraleedharan K.M., Illendula A., Avery M.A., Williamson J.S. (2005). Biocatalysis of the Antimalarial Artemisinin by *Mucor ramannianus* Strains. Pharm. Biol..

[17] Parshikov I.A., Muraleedharan K.M., Miriyala B., Avery M.A., Williamson J.S. (2004). Hydroxylation of 10-Deoxoartemisinin by *Cunninghamella elegans*. Nat. Prod..

[18] De Medeiros S.F., Avery M.A., Avery B., Leite S.G., Freitas A.C.C., Williamson J.S. (2002). Biotransformation of 10-deoxoartemisinin to its 7β-hydroxy derivative by *Mucor ramannianus*. Biotechnol. Lett..

[19] Parshikov I.A., Miriyala B., Avery M.A., Williamson J.S. (2004). Hydroxylation of 10-deoxoartemisinin to 15-hydroxy-10-deoxoartemisinin by *Aspergillus niger*. Biotechnol. Lett..

[20] Khalifa S.I., Baker J.K., Jung M., McChesney J.D., Hufford C.D. (1995). Microbial and Mammalian Metabolism Studies on the Semisynthetic Antimalarial, Deoxoartemisinin. Pharm. Res..

[21] Adachi T., Saito M., Sasaki J., Karasawa Y., Araki H., Hanada K., Omura S. (1993). Microbial Hydroxylation of (-)-Eburnamonine by *Mucor circinelloides* and *Streptomyces violens*. Chem. Pharm. Bull..

[22] Ma Y., Xie D., Wang Z.H., Dai J.-G., An X.-Q., Gu Z.-Y. (2015). Microbial transformation of glycyrrhetinic acid by *Cunninghamella blakesleeana*. Chin. J. Trad. Chin. Med..

[23] Dong T., Wu G.W., Wang X.N., Gao J.-M., Chen J.-G., Lee S.-S. (2010). Microbiological transformation of diosgenin by resting cells of filamentous fungus, *Cunninghamella echinulata* CGMCC 3.2716. J. Mol. Catal. B Enzym..

[24] Qin S., Zhou C.-L. (2004). Application of Microbial Transformationin Medicine Metabolization Model in vitro by *Cunninghammella Matruchot*. Strait Pharm..

[25] Weidner S., Goeke K., Trinks U., Traxler P., Ucci-Stoll K., Ghisalba O. (1999). Preparation of 4-(4′-Hydroxyanilino)-5-anilinophthalimide and 4, 5-Bis-(4′-hydroxyanilino)-phthalimide by Microbial Hydroxylation. Biosci. Biotechnol. Biochem..

[26] Lee I.S., ElSohly H.N., Croom E.M., Hufford C.D. (1989). Microbial metabolism studies of the antimalarial sesquiterpene artemisinin. J. Nat. Prod..

[27] Ye M., Han J., Tu G., An D., Guo D. (2005). Microbial hydroxylation of bufalin by Cunninghamella blakesleana and Mucor spinosus. J. Nat. Prod..

[28] Parshikov I.A., Muraleedharan K.M., Avery M.A., Williamson J.S. (2004). Transformation of artemisinin by *Cunninghamella elegans*. Appl. Microbiol. Biotechnol..

[29] Baydoun E., Ahmad M.S., Mehmood H., Ahmad M.S., Malik R., Smith C., Choudhary M.I. (2016). Microbial transformation of danazol with *Cunninghamella blakesleeana* and anti-cancer activity of danazol and its transformed products. Steroids.

[30] Sasaki J., Mizoue K., Morimoto S., Adachi T., Omura S. (1988). Microbial transformation of 6-O-methylerythromycin derivatives. J. Antibiot. Tokyo..

[31] Zhan Y.L., Wu Y.S., Xu F.F., Bai Y., Guan Y., Williamson J.S., Liu B. (2017). A novel dihydroxylated derivative of artemisinin from microbial transformation. Fitoterapia.

[32] Ma Y., Sun P., Zhao Y.F., Wang K., Chang X., Bai Y., Zhang D., Yang L. (2019). A Microbial Transformation Model for Simulating Mammal Metabolism of Artemisinin. Molecules.

[33] Betts R.E., Walters D.E., Rosazza J.P. (1974). Microbial Transformations of Antitumor Compounds. 1. Conversion of Acronycine to 9-Hydroxyacronycine by *Cunninghamella echinulata*. J. Med. Chem..

[34] Elmarakby S.A., Clark A.M., Baker J.K., Hufford C.D. (1986). Microbial Metabolism of Bornaprine, 3-(Diethylamino)propyl 2-Phenylbicyclo [2.2.1] heptane-2-carboxylate. Pharm. Sci..

